# Effect of Cocoa Butter and Sunflower Oil Supplementation on Performance, Immunoglobulin, and Antioxidant Vitamin Status of Rats

**DOI:** 10.1155/2014/606575

**Published:** 2014-07-16

**Authors:** Ebru Yıldırım, Miyase Çınar, İlkay Yalçınkaya, Hüsamettin Ekici, Nurgül Atmaca, Enes Güncüm

**Affiliations:** ^1^Pharmacology and Toxicology Department, Veterinary Faculty, Kirikkale University, 71451 Kirikkale, Turkey; ^2^Biochemistry Department, Veterinary Faculty, Kirikkale University, 71451 Kirikkale, Turkey; ^3^Animal Nutrition and Nutritional Diseases Department, Veterinary Faculty, Kirikkale University, 71451 Kirikkale, Turkey; ^4^Physiology Department, Veterinary Faculty, Kirikkale University, 71451 Kirikkale, Turkey

## Abstract

This study investigated the effects of cocoa butter and sunflower oil alone and in combination on performance, some biochemical parameters, immunoglobulin, and antioxidant vitamin status in Wistar rats. Forty-eight male rats were assigned to four groups, consisting of 12 rats with 3 replicates. Control received balanced rat diet without oil, cocoa butter group received 3.5% cocoa butter, sunflower oil group received 3.5% sunflower oil, the last group received 1.75% sunflower oil + 1.75% cocoa butter supplementation in the rat diet for 8 weeks. The total feed consumption in sunflower oil group was statistically lower than in the other groups. The serum creatinine level was decreased in cocoa butter group compared to control. Triglyceride and VLDL cholesterol levels were decreased in only sunflower oil and only cocoa butter groups as compared to control. The level of Ig M was statistically lower in cocoa butter and cocoa butter + sunflower oil groups than in control and sunflower oil groups. There were no statistically important difference in vitamin concentrations among trial groups. It was concluded that the supplementation of cocoa butter in diet decreased Ig M level, while the supplementation of cocoa butter and sunflower oil alone decreased the triglyceride and VLDL cholesterol levels.

## 1. Introduction 

Cocoa is a product derived from the beans of* Theobroma cocoa* plant and mostly found in West Africa, Central and South Africa, and tropical areas of Asia [1]. Although cocoa is thought to be a luxury agent, it has recently been considered a therapeutic product, because it contains important substances such as fats (cocoa butter), antioxidants (flavonoids, epicatechin, catechin, and procyanids), nitrogenous compounds, and minerals [2, 3]. Most of the polyphenols show an in vitro antioxidant effect [4], therefore helping to protect the body against reactive oxygen species. The antioxidant products found in cocoa inhibit the oxidation of low density lipoprotein (LDL-cholesterol); thus, they show a protective effect against heart disease [1]. These phenolic agents confer cocoa resistance to peroxidation and have an antioxidant and immune regulatory effect [2].

Cocoa butter is also extracted from the* Theobroma cocoa* seeds [5]. The bioavailability of cocoa butter is poor, so its absorption is weak compared to other oils [1]. Cocoa butter contains 33% oleic acid, 25% palmitic acid, and 33% stearic acid. Oleic acid has beneficial effects on lipid levels [3]. Chen et al. [6] showed that the fatty acid recovered from thoracic lymph and the absorption of cholesterol were lower in rats given cocoa butter when compared with rats receiving corn oil. Apgar et al. [7], in a supplemented diet fed to rats over 2 weeks, showed that cocoa butter had lower digestibility coefficients compared to corn oil. Porsgaard and Høy [8] also detected a lower triacylglycerol level in the lymph of rats that received cocoa butter compared to olive and corn oil. Cocoa butter contains stearic acid that is less absorbed than other fatty acids. Thus, the bioavailability of cocoa butter is low [1]. In studies conducted on rats, cocoa butter has been shown to lower cholesterol [9, 10] and triglyceride levels [10]. Sunflower oil is a well-known dietary component [11] that has also been shown to have triglyceride lowering effect [12].

There is a close relationship between the adipose and immune system [13] and vitamins [14]. Wander et al. [15] showed that in older dogs, the dietary fatty acid ratios in the diet affect the metabolism of the immune system, lipid peroxidation, and vitamin E concentrations. Beketova et al. [16] suggested that an excessive or reduced consumption of fats can induce polyhypovitaminosis. There are many studies that show the immunomodulatory effects of cocoa [17–20], also cocoa butter has been shown to modulate immune response [21–23] and antioxidant enzyme systems [24]. However, to our knowledge, no study has been conducted to determine the effect of cocoa butter or sunflower oil, alone and combined, on antioxidant vitamins (vitamins A, C, E) and immunoglobulins. Therefore, the aim of this study was to investigate the effects of cocoa butter or sunflower oil alone and in combination on the growth performance, some biochemical parameters, and immune and antioxidant vitamin status in rat.

## 2. Materials and Methods

### 2.1. Animals and Experimental Design

Forty-eight male Wistar rats aged 5 to 6 months were used in this study. The animal care and use protocol was reviewed and approved by the Ethics Committee of Kırıkkale University (2012/09). The rats were assigned to four groups, consisting of 12 rats with 3 replicates. The diet for the four rat groups was calculated to contain adequate levels of required nutrients. The control group received a rat diet without oil. The cocoa butter group received 3.5% cocoa butter, the sunflower oil group received 3.5% sunflower oil, and the last group received 1.75% sunflower oil + 1.75% cocoa butter supplementation in rat diet.

The ingredients and chemical composition of the rat diet are presented in Table 1. The basal diet was balanced with a metabolic energy 2600 kcal/kg, and 23% crude protein. The rats were maintained under controlled conditions with food and water being offered* ad libitum*. During the experiment, there was a 12 h light/12 h dark cycle.

### 2.2. Determination of Performance Parameters and Relative Organ Weights

The rats were randomly assigned according to their initial body weights. At the end of the experimental period, the body weight (BW), body weight gain (BWG), and the total feed consumption (TFC) of rats were recorded. The liver and kidney were removed, weighed, and expressed as relative organ weights (g/100 g live weight). The study ended at the 8th week of the experiment.

### 2.3. Sample Collection and Analysis

At the end of the 8 weeks, rats were anesthetized with sodium thiopental (50 mg/kg) and blood samples were collected into heparinized or serum test tubes from the heart of the rats. The blood samples were centrifuged at 3000 rpm for 10 min to separate the plasma and serum. The activities of aspartate aminotransferase (AST), alanine aminotransferase (ALT), and alkaline phosphatase (ALP) and the levels of total protein, albumin, creatinine, uric acid, total bilirubin, total cholesterol, high density lipoprotein (HDL cholesterol), triglyceride, glucose, and calcium in the serum were determined by an autoanalyzer (Beckman Coulter AU680, USA) using commercial test kits (Beckman, USA). Low density lipoprotein (LDL cholesterol) and very low density lipoprotein (VLDL cholesterol) were calculated from primary measurements using Friedewald's empirical formula [25].

The levels of immunoglobulin G (Ig G) and immunoglobulin M (Ig M) were also measured in the plasma by an ELISA reader (SIRIO S, Italy) using ELISA commercial test kits (Cusabio, rat immunoglobulin M, ELISA kit, catalogue number CSB-E07978r; Cusabio, rat immunoglobulin G, ELISA kit, catalogue number CSB-E079981r, China) with a double reading.

#### 2.3.1. Determination of Vitamin A

Plasma vitamin A concentrations were determined as described by Suzuki and Katoh [26].

*Reagents*. Absolute ethanol (Merck 1.00983), butylated hydroxytoluene (BHT), (Merck 817074), and n-hexane (Merck 1.04368) were used.

Briefly, 0.5 mL of ethanol containing BHT (20 *µ*g/ml) was added to the 0.5 mL of plasma, and then 1.5 mL of n-hexane was added. Shake the tube by hand for 10 min and centrifuge the tube at 2000 rpm 10 min, +4°C. After centrifugation, the upper hexane layer from each tube was placed into test tubes. The vitamin A absorbances were measured by a spectrophotometer at 325 nm aqainst n-hexane.

#### 2.3.2. Determination of Vitamin C

Plasma vitamin C concentrations were analyzed by the method given by Haag [27].

*Reagents*. Trichloroacetic acid (Merck 1.00810), sulphuric acid (H_2_SO_4_) (Merck 1.00713), 2,4-dinitrophenylhydrazin (Aldrich D 19.930-3), copper (II) sulphate (Carlo Erba CASN 7758-99-8), thiourea (Sigma T8656), and L-ascorbic acid (Riedel 33034) were used. Basic solution is as follows: 2 g of 2,4-dinitrophenylhydrazin was dissolved in 100 mL 4.5 M H_2_SO_4_. These solutions were kept in a dark volumetric flask at +4°C temperature for 1 night in a refrigerator. After incubation, it was filtered into 100 mL volumetric flask.

Color reagent was prepared by the combination of thiourea 5%, CuSO_4_·5H_2_O 0.6% with the basic solution (5/5/100 V,V/V).

Briefly, 0.5 mL of plasma sample and 1 mL of TCA 6% solution were combined, mixed, and centrifuged at 3000 rpm/15 min. +4°C, and then 500 *μ*L of supernatants, standard (%10 *μ*g/mL ascorbic acid), and blank (TCA 6%) were transferred into different test tubes. An equal volume of (200 *μ*L) color reagent was added to each tube. These tubes were kept in water baths at 38°C for 4 hours. After this incubation, all the test tubes were cooled in an ice bath and treated with 1 mL of 12 M H_2_SO_4_ with constant stirring. As a result, a colored solution was obtained, and absorbance was measured at 520 nm against the blank.

#### 2.3.3. Determination of Vitamin E

The determination of *α*-tocopherol was carried out using the method by Martinek [28].

*Reagents*. *α*-Tocopherol (Sigma 3251), absolute ethanol (Merck 1.00983), 2,4,6-tripyridyl-s-triazine (TPTZ, Sigma T 1253), ferric chloride (Merck 1.03943), xylene (Merck 1.08685), and n-propanol (Merck 1.00997) were used.

0.5 mL of plasma, standard solution (200 mg/mL *α*-tocopherol), and blank (distilled water) were taken into different glass tubes. An equal volume of 0.5 mL absolute ethanol was added to the sample and blank, while 0.5 mL distilled water was added to the standard. To each tube add 0.5 mL xylene. Shake vigorously for at least 30 sec and subject it to centrifugation for 5 min at 2500 rpm, 4°C. After centrifugation, 250 *μ*L of the upper xylene layer from each tube was transferred to test tubes. To each test tube, add 250 *μ*L of TPTZ solution and mix. The samples were measured at 460 nm against the blank within 4 min. Then 50 *μ*L ferric chloride solution was added to each tube, and then the absorbances were measured at 600 nm against the blank.

### 2.4. Statistical Analysis

Data processing was performed with the SPSS 15.0 (SPSS, Inc., Chicago, IL, USA). The normality of all data was assessed by the Shapiro-Wilk test. The relative weight of kidney, the activities of AST and ALT, and the levels of albumin, creatinine, calcium (Ca), total bilirubin, and Ig M were tested using the Kruskal-Wallis test followed by the Mann-Whitney *U* test with Bonferroni adjustment to determine which of the four groups differed from the others. The other parameters (parametric) were analyzed by one-way ANOVA test. When the *F* values were significant, Duncan's Multiple Range test was performed. The differences were considered significant when *P* < 0.05 in the one-way ANOVA and Kruskal-Wallis tests, and *P* < 0.0083 in the Mann-Whitney *U* test with a Bonferroni adjustment.

## 3. Results 

### 3.1. Performance Parameters

The initial and final BW(s) of the animals were determined and expressed as grams. There was no difference between groups in terms of BW (*P* > 0.05). The initial and final BW(s), BWG, and TFC of animals are shown in Table 2. No differences were detected in the BWG of the animals during the trial period among experimental groups. However, the TFC was lower in sunflower oil group when compared with the other groups (*P* < 0.01) (Table 2). No differences were observed among groups in terms of the relative weights of liver and kidney (Table 3).

### 3.2. Biochemical Parameters

The effects of sunflower oil and cocoa butter alone and combined on serum lipid profile are presented in Table 4. In comparison with the control group, the serum triglyceride and VLDL cholesterol levels were significantly decreased in only the cocoa butter and sunflower oil groups (*P* < 0.01). The effects of sunflower oil and cocoa butter alone and combined on some serum enzyme activities and metabolite levels are shown in Table 5. The serum creatinine was statistically reduced in the cocoa butter group compared with the control group (*P* < 0.01). Neither the sunflower oil nor the cocoa butter caused changes in the other biochemical parameters.

### 3.3. Ig G and Ig M Parameters

Although there were no statistically significant changes in terms of Ig G values between trial groups, an insignificant increase was seen in the cocoa butter group in comparison to the control group. The Ig M levels were significantly decreased (*P* < 0.001) in cocoa butter alone and sunflower oil + cocoa butter group as compared to the control and only sunflower oil group. The Ig G and Ig M results of trial group are given in Table 6.

### 3.4. Vitamins A, C, and E Parameters

The concentrations of vitamins A, C, and E did not differ statistically among the four trial groups (*P* > 0.05). However, the concentration of vitamin A insignificantly decreased in cocoa butter group as compared to the control group. The average vitamin concentrations of trial groups are presented in Table 7.

## 4. **Discussion**

One of the most important supplies of energy in a diet comes from fats and oils; each gram of oil or fat provides 9 Kcal energy [29] and they have many important functions in living organisms [30]. Sunflower oil and cocoa butter can be a good choice to be included in a diet. Kritchevsky et al. [31] fed rats (187 g, or 260 g average weight) on 14% fat (corn oil, coconut oil, palm kernel oil, and cocoa butter) for 21 days and showed that there was no difference in weight gain among trial groups in rats with higher average weight, with the weight gain being lower in rats with lesser average weight that were given cocoa butter. Dei et al. [32] found no difference between the broilers fed on 30, 60, and 90 mg/kg cocoa butter in terms of BWG and TFC. Morrissey et al. [9] showed that graded levels of cocoa butter (0, 10, and 30%) did not cause a deleterious effect on the growth of rats. Also in our study, the BWG did not change among groups, but the TFC was reduced in the group that was given sunflower oil. These findings show that cocoa butter or sunflower oil alone and in combination have no negative effects on performance. Consequently, they can be defined as oils that can be safely included in a diet.

In a study by Ahmad et al. [29] the weight of liver was significantly higher in rats given 7% fish oil and soybean oil for 7 weeks. Similarly Astorg and Levillain [33] found that transmonoenes and erucic acid induce an increase in liver weight. Imofidon and Okunrobo [34] showed that the increase in liver weights of rats that received a 10% palm and groundnut oil supplemented diet was accompanied with a mild inflammation in the liver. Kritchevsky et al. [31] detected that 187 g rats fed on 14% cocoa butter for 21 days had a lower relative liver weight compared to those rats that received corn, and palm kernel oil, and similar to our study no differences were seen among rats weighing 260 g and fed on 14% fats for 21 days. These discrepancies among studies are attributed to the different fat or oil sources, the dietary fat percentage used in the diet, the age, and the weight of the rats. In accordance with our study, Morissey et al. [9] showed that there were no pathological abnormalities due to the addition of cocoa butter up to 30% for 90 days. This may explain the reason why there is no difference in terms of the liver and kidney weights in our study.

The total cholesterol, HDL cholesterol, and triglycerides are known as the lipid profile [35] and are found to be significant determinants in metabolic disorders such as hypertension, heart disease, diabetes, and dyslipidemia [36]. In accordance with the results of Garg and Blake [12], the group receiving sunflower oil had lower triglyceride levels. Sunflower oil is rich in omega 6 polyunsaturated fatty acids (PUFA) [37]. PUFA thought to reduce the hepatic synthesis of fatty acids, which decreases the level of triglycerides in the liver [38]. Although Garg and Blake [12] showed that HDL cholesterol levels were lower in sunflower oil given rats, no differences were observed in the level of HDL cholesterol between the groups in the current study. Similar to our study, Lee et al. [39] stated that sunflower oil did not cause a significant change in HDL-cholesterol levels in human. Öz and Kurtoğlu [40] reported that there were no significant changes in the triglyceride levels of rats given sunflower oil. Morissey et al. [9] studied the graded levels of cocoa butter (10 and 30%) and showed the cholesterol lowering effect. Kiitchevsky et al. [10] showed that 10% cocoa butter in rat diet lowered the liver cholesterol and triglyceride levels when compared to the groups given 10% corn oil, palm kernel oil, and coconut oil; on the other hand, serum cholesterol levels were found to be similar in the groups given cocoa butter or corn oil and they were lower than in the group receiving palm kernel oil. No change was detected in the level of cholesterol in our study. The reduction in the triglyceride level of cocoa butter group may be attributed to the stearic acid found in cocoa butter. Stearic acid is more insoluble than other fatty acids, therefore poorly absorbed and cannot enter the body [41].

Increased plasma triglyceride and VLDL cholesterol levels are due to the increase in hepatic overproduction of large triglyceride-enriched VLDL cholesterol particles. Dyslipidemia is characterized by elevated VLDL cholesterol and triglyceride [42]. Reducing the level of plasma triglycerides is important because it can assist in the development of new or improved pharmacological approaches to treating hypertriglyceridemia [43]. In our study, the level of VLDL cholesterol was decreased only in the groups receiving cocoa butter or sunflower oil. The decrease in VLDL cholesterol can result from a reduction of the rate of triglyceride synthesis, an enhancement of triglyceride removal, or a combination of these effects [44].

Nazari et al. [45] showed that the essential oil of* Satureja khuzestanica* reduced the level of creatinine; similarly in our study creatinine was reduced by the supplementation of cocoa butter alone. This result showed that cocoa butter may also have beneficial effects on renal function. In fact, dark chocolate has been found to increase renal medullary oxygenation [46]. This may be related to the antioxidant effect of cocoa butter. Further studies need to be conducted to determine the underlying mechanism.

The changes in the serum enzyme activities are important indicators in the early diagnosis for diseases or tissue damage due to a toxic substance. Serums ALP, ALT, and AST enzyme activities were among the most important markers for the biochemical analysis in liver [47]. Mohammed and Luka [48] showed that serums ALT and ALP activities were significantly decreased in rats that received coconut and palm kernel oil; however, no toxic effects were found in the liver. As there were no significant changes in the activities of AST, ALP, and ALT, cocoa butter (3.5%) was thought not to be hazardous to the liver.

There are studies showing the effects of dietary fatty acids on immune system [49, 50]. For example, Fritsche et al. [50] showed that animals fed with corn, canola, flaxseed, or fish oil for three weeks had higher Ig M titers. This indicates that the dietary fats can also affect the immunoglobulin levels. In our study, the level of Ig G did not differ between the groups while the Ig M levels were significantly decreased in the group given cocoa butter. Ramiro-Puig et al. [17] demonstrated that the serums Ig A, M, and G concentrations were decreased in a 10% cocoa enriched diet. Pérez-Berezo et al. [19] also showed that serums Ig M and G concentration was reduced in rats given cocoa, suggesting that the downregulating effects on immunoglobulin can reveal a beneficial effect in hypersensitivity and autoimmunity.

Stahelin et al. [51] showed a correlation between the concentration of plasma lipid and vitamins A and E. In our study, the triglyceride and VLDL cholesterol levels were decreased only in the sunflower oil and cocoa butter given group but the amount of vitamin E did not change between groups. However, the amount of vitamin A was insignificantly decreased in only the sunflower oil and cocoa butter groups, indicating a relationship between dietary lipids and vitamins. Beketova et al. [16] studied the effect of a low (1%) and high (31%) dietary fat content (sunflower-seed oil and lard 1 : 1) on the vitamins A and E status of rats and showed that an excessive intake of fat and vitamin E for 6 weeks did not influence the content of blood plasma vitamin E similar to our study, but they noted an increase in the concentration of plasma vitamin A.

## 5. Conclusion

In conclusion, the dietary supplementation of cocoa butter and sunflower oil alone for 8 weeks was found to be more effective in some lipid profile parameters (triglyceride and VLDL cholesterol) than the combined treatment. The downregulating effect on the level of Ig M in cocoa butter groups suggested that the immune modulatory effect of cocoa may be partially attributed to the cocoa butter.

## Figures and Tables

**Table 1:** The composition of a balanced rat diet (%).

Ingredients	
Corn	42
Barley	15
Sunflower meal	11.5
Meat meal	24.5
Wheat bran	3.85
Alfalfa meal	2.5
Oil	—
Salt	0.30
Vitamin mineral mix^1^	0.35

^1^Vitamin contribution to diet (mg/kg): thiamin, 5; riboflavin, 6; pyridoxine, 6; nicotinic acid, 30; pantothenate, 15; folic acid, 2; phylloquinone, 0.75; biotin, 0.2; cyanocobalamin, 0.025; all-*trans*-retinyl palmitate (500,000 IU/g), 8; all-*rac*-atocopheryl acetate (500 IU/g), 150; and cholecalciferol (400,000 IU/g), 2.5. Mineral contribution to diet (mg/kg): Ca, 5000; P, 1561; K, 3600; Na, 1019; Cl, 1571; S, 300; I, 0.2; Fe, 35; Mg, 507; Zn, 30; Cu, 6; Mn, 10; Mo, 0.15; Se, 0.15; Cr, 1; Si, 5; F, 1; Ni, 0.5; B, 0.5; Li, 0.1; and V, 0.1.

**Table 2:** Effects of sunflower oil and cocoa butter, alone or in combination, on initial and final body weight, body weight gain, and total feed consumption in rats (g).

Parameters	Control (*n*: 12)	Sunflower oil (*n*: 12)	Cocoa butter (*n*: 11)	Sunflower oil + cocoa butter (*n*: 12)	*P* value
Initial BW	279.33 ± 3.71	281.75 ± 7.52	274.83 ± 9.29	286.42 ± 8.21	NS
Final BW	303.50 ± 5.85	295.83 ± 7.55	304.18 ± 9.82	316.42 ± 9.70	NS
BWG	24.17 ± 4.45	14.08 ± 9.77	28.45 ± 15.51	30.00 ± 12.47	NS
TFC	1468.67 ± 56.86^a^	1245.42 ± 28.06^b^	1642.31 ± 62.45^a^	1448.67 ± 71.20^a^	0.008

The mean values within the same row with different superscripts letter ^a,b^ are significantly different. Data were expressed as mean ± standard error. NS: not significant and *n*: number of animals.

**Table 3:** Effects of sunflower oil and cocoa butter, alone or in combination, on the relative organ weights (g/100 g live weight) in rats (g).

Parameters	Control (*n*: 12)	Sunflower oil (*n*: 12)	Cocoa butter (*n*: 11)	Sunflower oil + cocoa butter (*n*: 12)	*P* value
Liver	3.09 ± 0.06	3.01 ± 0.11	3.24 ± 0.09	2.98 ± 0.07	NS
Kidney	0.71	0.73	0.73	0.73	NS

The parameters of liver were expressed as mean ± standard error while kidney parameters were expressed as median. There were no differences between trial groups (*P* > 0.05). NS: not significant and *n*: number of animals.

**Table 4:** Effects of sunflower oil and cocoa butter, alone or in combination, on serum lipid levels in rats.

Parameters	Control (*n*: 12)	Sunflower oil (*n*: 12)	Cocoa butter (*n*: 11)	Sunflower oil + cocoa butter (*n*: 12)	*P* value
Triglyceride (mg/dL)	49.17 ± 3.60^a^	35.83 ± 2.69^b^	32.91 ± 3.37^b^	40.67 ± 2.61^ab^	0.003
Total Cholesterol (mg/dL)	97.83 ± 4.69	90.58 ± 4.67	98.64 ± 5.58	104.67 ± 4.91	NS
HDL cholesterol (mg/dL)	62.59 ± 3.04	61.82 ± 3.09	66.45 ± 3.31	70.80 ± 3.31	NS
LDL cholesterol (mg/dL)	25.42 ± 2.21	21.58 ± 1.56	25.73 ± 2.25	25.75 ± 2.13	NS
VLDL Cholesterol (mg/dL)	9.83 ± 0.69^a^	7.08 ± 0.56^b^	6.54 ± 0.69^b^	8.08 ± 0.53^ab^	0.003

Mean values within the same row with different superscripts letter ^a,b^ are significantly different. NS: Not significant and *n*: number of animals.

**Table 5:** Effects of sunflower oil and cocoa butter alone, or in combination, on some serum enzymes activity and metabolites levels in rats.

Parameters	Control (*n*: 12)	Sunflower oil (*n*: 12)	Cocoa butter (*n*: 11)	Sunflower oil + cocoa butter (*n*: 12)	*P* value
ALP (U/L)	312.58 ± 31.39	268.94 ± 39.76	274.83 ± 32.63	325.86 ± 58.34	NS
AST (U/L)	194.00	151.00	200.00	215.50	NS
ALT (U/L)	70.00	61.00	64.50	63.50	NS
Glucose (mg/dL)	137.08 ± 7.86	120.33 ± 15.57	132.09 ± 15.08	120.50 ± 8.50	NS
Total bilirubin (mg/dL)	0.13	0.16	0.14	0.13	NS
Uric acid (mg/dL)	2.17 ± 0.15	2.83 ± 0.28	3.05 ± 0.37	2.52 ± 0.29	NS
Creatinine (mg/dL)	0.53^a^	0.48^ab^	0.46^b^	0.48^ab^	0.004
Total protein (g/dL)	7.66 ± 0.11	7.63 ± 0.15	7.39 ± 0.16	7.55 ± 0.13	NS
Albumin (g/dL)	3.32	3.29	3.23	3.28	NS
Calcium (mg/dL)	10.80	10.90	11.05	11.05	NS

Data within the same row with different superscripts letter ^a,b^ are significantly different. Parametric data were expressed as mean ± standard error. Nonparametric values were expressed as median. NS: not significant and *n*: number of animals.

**Table 6:** Effects of sunflower oil and cocoa butter, alone or in combination, on Ig G and Ig M levels in rats.

Parameters	Control	Sunflower oil group	Cocoa butter group	Sunflower oil + cocoa butter group	*P* value
Ig G (ng/mL)	1414.66 ± 154.32 (*n*: 11)	1522.79 ± 143.92 (*n*: 10)	1789.97 ± 162.17 (*n*: 11)	1717.47 ± 212.08 (*n*: 11)	NS
Ig M (ng/mL)	378.95^a^ (*n*: 12)	387.96^a^ (*n*: 10)	139.40^b^ (*n*: 11)	125.96^b^ (*n*: 11)	0.000

Data within the same row with different superscripts letter ^a,b^ are significantly different. In terms of Ig G, the data were expressed as mean ± standard error. Ig M values were expressed as median. NS: Not significant and *n*: number of animals.

**Table 7:** Effects of sunflower oil and cocoa butter, alone or in combination, on plasma vitamins A, C, and E concentrations in rats.

Parameters	Control	Sunflower oil	Cocoa butter	Sunflower oil + cocoa butter	*P* value
Vitamin A (*μ*g/dL)	68.22 ± 6.63 (*n*: 6)	55.46 ± 5.90 (*n*: 6)	45.67 ± 5.31 (*n*: 6)	58.23 ± 12.06 (*n*: 6)	NS
Vitamin C (*μ*g/mL)	10.29 ± 0.91 (*n*: 9)	10.71 ± 0.67 (*n*: 9)	12.39 ± 0.65 (*n*: 9)	11.50 ± 0.59 (*n*: 9)	NS
Vitamin E (mg/dL)	0.83 ± 0.20 (*n*: 9)	0.82 ± 0.09 (*n*: 9)	0.70 ± 0.07 (*n*: 9)	0.87 ± 0.08 (*n*: 9)	NS

Data were expressed as mean ± standard error. There were no differences between trial groups (*P* > 0.05). NS: not significant and *n*: number of animals.
